# Adult Hippocampal Neurogenesis Is a Developmental Process Involved in Cognitive Development

**DOI:** 10.3389/fnins.2019.00159

**Published:** 2019-03-06

**Authors:** Mikhail V. Semënov

**Affiliations:** ^1^Bedford Division, New England Geriatric Research Education and Clinical Center, Edith Nourse Rogers Memorial Veterans Hospital, Bedford, MA, United States; ^2^The Department of Pathology and Laboratory Medicine, Boston University School of Medicine, Boston, MA, United States

**Keywords:** adult neurogenesis, subgranular zone, neural stem cells, radial glia like cells, hippocampus, cognitive neurogenesis, neural transit cells, developmental processes

All processes in the body of animals can be divided into three groups. Developmental processes are responsible for the formation of tissues and organs via the addition of new cells and their differentiation into the proper cell types to ensure correct organ and tissue functionality. Adult processes are responsible for the maintenance of organ and tissue functionality, in part via the replacement of burnout cells. And aging processes that are characterized by the decline of tissue and organ functionality, often coinciding with cell loss. There is no sharp boundary between these processes and they smoothly transfer from one into another. Adult hippocampal neurogenesis (AHN) in the mammalian brain appears to be a late developmental process supported by neural transit cells (NTCs) that never advances into the adult process stage (Bonfanti, 2016; Lipp and Bonfanti, 2016).

Postnatal hippocampal neurogenesis was discovered by Altman and Das in laboratory rats in 1965 (Altman and Das, 1965). They showed that neurogenesis could be observed in rat brains up to the age of 8 months. The term “adult neurogenesis” was introduced by Goldman and Nottebohm in 1983 to describe neurogenesis in the adult female canary brain (Goldman and Nottebohm, 1983). Now this term is ubiquitously used to describe hippocampal neurogenesis in the adult mammalian brain. According to current understanding, new neurons in the hippocampus are produced by neural stem cells (NSC) located in the subgranular zone (SGZ) in the dentate gyrus (DG) (Balu and Lucki, 2009; Ming and Song, 2011; Gonçalves et al., 2016). However, the comparison of adult neurogenesis with other adult stem cell-supported processes shows that AHN is a profoundly different process.

AHN is best studied in mice and therefore, we will use mostly data obtained in mice in this opinion paper. Laboratory mice live in a fairly unchanging environment. The mice often live in the same cages, same rooms, same day/night schedule, with the same cage partners all their lives. This should result in steady neuronal activity in the mouse brain that, as we might expect, would require a stable rate of granule cell replacement. And yet, the rate of neurogenesis decreases exponentially during the entire adult mouse life and never reaches a steady rate (Ben Abdallah et al., 2010; Smith and Semënov, 2019). The decrease of AHN with age was first noticed by Altman and Das in rats (Altman and Das, 1965). Later this decrease was reported in many other mammalian species (Seki and Arai, 1995; Kuhn et al., 1996; Drapeau and Nora Abrous, 2008; Morgenstern et al., 2008; Lee et al., 2012; Apple et al., 2017; Mosher and Schaffer, 2017; Smith et al., 2017) showing that it is an intrinsic feature of AHN. This is in contrast to adult renewal processes in blood, epidermis, and small intestine epithelia which proceed at a steady rate during the entire adult mouse life.

Somatic cells in the epidermis and small intestine epithelia are steadily replaced multiple times during the mouse lifespan. To achieve this, new somatic cells are added to the epidermis near the basal cell layer and to the intestinal epithelia near the base of the crypt. Over time these cells move toward the external layer of the epidermis or the tip of the small intestine villi where they undergo programmed cell death (PCD). Thus, the site of new cell addition and the site of PCD are located at the opposite side of these tissues allowing steady renewal of all cells. In contrast, new neurons are added to the inner surface of the granule cell layer (Morgenstern et al., 2008; Mathews et al., 2010) and PCD is also observed in the same place (Biebl et al., 2000; Sun et al., 2004). The rate of PCD decreases with the decrease in the AHN rate (Sun et al., 2004), showing that it is newly added neurons and neural precursors undergoing PCD. New cell addition and PCD at the same location show that AHN is not capable of supporting the continuous renewal of all neurons in the granular cell layer.

From the age of 30 days to the age of 2.5 years, about 1.5 million neural precursors are produced in the SGZ (Smith and Semënov, 2019). About 80% or more of these precursors will differentiate into new neurons (Figure 4D in Encinas et al., 2011). This number of new neurons is sufficient to replace all neurons in the DG about two times during the mouse lifespan. However, it appears that most neurons produced during brain development in the DG are preserved during the entire mouse lifespan and new neurons produced by AHN are added to the inner surface of the granule cell layer (Ninkovic et al., 2007; Imayoshi et al., 2008). The measured extent of this addition varies from about 10% (Imayoshi et al., 2008), to 7% (Ninkovic et al., 2007) or 10,000 new cells (about 2%) (Lagace et al., 2007). Thus, out of 1.5 million new neural progenitors, only 50,000 or so are able to incorporate into the DG as new neurons. Such large attrition, 97%, clearly shows that new neurons produced by AHN are not destined to replace old granular cells in the DG but rather provide a continuous supply of new neurons to complete the functional development of the DG.

The idea that adult neurogenesis is supported by stem cells located in or nearby the SGZ comes from studies of blood, skin, and intestinal epithelia renewal. In these tissues, stem cells serve as the ultimate source of all new cells. Therefore, when RGL cells were identified as the source of new neurons and glial cells in the DG, they had been proposed to be NSCs (Nacher et al., 2001; Seri et al., 2001). The presence of NSCs in the SGZ was questioned less than a half year after their identification. When performing *in vitro* clonal analysis of stem cells in the SGZ and the subventricular zone (SVZ), Seaberg and van der Kooy came to a conclusion that the SGZ contains no NCSs but rather restricted neuronal progenitors with “very limited self-renewal ability” (Seaberg and van der Kooy, 2002). Due to a possibility that the condition for stem cell cultivation used in this study was not optimal for stem cells from the SGZ, this conclusion was not appropriately considered at the time.

Stem cell is a functional term and only cells that possess stem cell characteristics could be named as such (Potten and Loeffler, 1990). Stem cells are defined by four key characteristics (Potten and Loeffler, 1990).

1. Self-maintenance is the defining characteristic among them. Only cells that proliferate and are able to maintain their identity can be named as stem cells. Population analysis of RGL cell proliferation shows that RGL cells divide about three times in <7 days and after that convert into astrocytes (Encinas et al., 2011). Continued live imaging shows that RGL cells divide 2–3 times in less than a week and after that lose markers of RGL cells (Pilz et al., 2018). The number of RGL cells in the mouse brain decreases 100 times from the age of 3 weeks to the age of 24 months (Encinas et al., 2011), showing that at least 99% of RGL cells could not maintain themselves or their identity. This is in contrast to epidermal and intestinal stem cells that maintain their number and activity throughout the life of the mouse (Stern and Bickenbach, 2007; Giangreco et al., 2008; Nalapareddy et al., 2017). Thus, RGL cells possess no self-maintenance ability.
2. The ability to produce a large family of differentiated functional cells. Population analysis of RGL cell proliferation shows that each RGL cell produces only about a dozen progenitors (Encinas et al., 2011). Continued live imaging shows that each RGL cell, on average, produce 12 progenies in about 2 weeks (Pilz et al., 2018). Thus, RGL cells do not have the ability to produce a large family of differentiated functional cells.
3. The ability to regenerate tissue following injury. The presence of stem cells in skin and intestinal epithelia is clearly manifested by wound healing, in bones by healing of fractures, and in blood by reconstituting blood loss due to bleeding or blood donation. All of these healing processes are apparent and unambiguously show the presence of stem cells in these tissues (Ge and Fuchs, 2018). On the other hand, there is no evidence demonstrating that the damaged DG can self-repair. In that respect, the DG is similar to other parts of the adult mammalian brain that are not able to regenerate after the damage. Thus, RGL cells show no ability to regenerate the DG following injury.
4. The undifferentiated state. RGL cells are highly differentiated cells with complex morphology; they contain radial processes that are about 60–80 um in length ending by elaborate arborization (Gebara et al., 2016).

Thus, RGL cells have none of defining characteristics of stem cells. At the same time, they have all properties expected from NTCs. They are able to produce a limited number of progenitors during a limited time, and in the process, lose their identity and ability to proliferate. In addition, Potten and Loeffler predict that transit cells in the absence of stem cells could not maintain their population and must gradually disappear. The number of RGL cells decreases about 100 fold from the age of 3 weeks to the age of 24 months (Encinas et al., 2011), confirming that RGL cells have this characteristic of NTCs. Stem cells are responsible for the replenishment of the transit cell population and maintain it at the stable level (Potten and Loeffler, 1990). Therefore, the decrease of RGL cell numbers also indicates that the SGZ contains no active NSCs that are able to produce replacements for used up RGL cells.

While AHN is different from the adult stem cell driven tissue renewal processes, it has all the hallmarks of late developmental processes. At the end of development, embryonic stem cells produce the last batch of transit cells (TCs). These TCs in turn produce the last batch of somatic cell precursors. These precursors migrate to the sites of integration and try to integrate into the forming structures. The majority of these cells undergo PCD, and only a small fraction of them successfully integrate because the organs and tissues are almost completely formed by this time. Cell overproduction is required in order to be certain that organs and tissues are formed with the proper size and cell composition to be fully functional. This overproduction is very characteristic of the nervous system where many new neurons and neuronal connections are eliminated at the end of development. The major characteristics of these late developmental processes include: the decline of new precursor production with time; cell addition being directed at the completion of organ or tissue formation not at the replacement of burned out cells; new cells mostly undergo PCD; and PCD is observed at the site of new cell incorporation. AHN has all these characteristics.

Late developmental processes are usually quick and do not extend into the adulthood. The extension of AHN that often spans the entire life of mammals, the use of very specialized RGL cells, the complex regulation of RGL cell proliferation that allows them to persist for a long time in an inactive state, and the response of AHN to changes in the environment all show that AHN cannot be regarded as a simple extension of juvenile developmental processes into the adult age (Bonfanti, 2016; Lipp and Bonfanti, 2016) but rather that it is a distinct stage in the DG development.

AHN is a developmental process and therefore it ought to support postnatal brain development. There is practically only one developmental process that occurs in the postnatal mammalian brain and it is cognitive development. The innate cognitive abilities of mammalian brains, including humans, are limited to a number of reflexes and brain cognitive development occurs mostly after birth in direct interaction with the environment. Newborns show a remarkable rate of new cognitive skills acquisition. With age, the rate decreases through childhood, adolescence and adulthood and it can become unnoticeable in the elderly. Thus, cognitive development follows the neurogenesis dynamics from the highest rate in youth and follows a steady decline with age. Anti-cancer systemic chemotherapy and cranial radiation therapy leads to cognitive impairment. The most severe effect is observed in children, especially young children (Mulhern et al., 2004, 2005; Pendergrass et al., 2018). Experiments in mice show that anti-cancer therapy decreases adult neurogenesis and causes cognitive impairment in mice (Rola et al., 2004; Dietrich et al., 2015; Rendeiro et al., 2016). Thus, cognitive impairment is more severe in young children who have a higher rate of adult neurogenesis (Monje and Dietrich, 2012). Manipulations of adult neurogenesis in mice and other experimental animals show that adult neurogenesis is implicated in cognitive functions of the brain (Couillard-Despres et al., 2011; Oomen et al., 2014; Costa et al., 2015). There is also data showing that some human cognitive development diseases are associated with decreased adult neurogenesis [reviewed in (Bowers and Jessberger, 2016)]. Thus, it is plausible that adult neurogenesis plays a role in the cognitive development of the mammalian brain. To distinguish this role of AHN, we are proposing to name it “cognitive neurogenesis.”

The use of laboratory mice to study the role of cognitive neurogenesis has conceptual limitations. Laboratory mice are not exposed to practically any challenges that wild mice are, therefore, their cognitive development remains dismal. Any challenge, for example a cognitive test, enriched environment, running wheel, and so on, are unfamiliar to the mice and require expansion of their cognitive skills. The acquisition of new cognitive skills affects neurogenesis. Thus, almost any test will show some dependence or an effect on neurogenesis. However, it is impossible to distinguish if the effect is due to the acquisition of new cognitive skills or the performance of the task. This conclusion can be illustrated by the role of AHN in the spatial memory formation. It is well-established from experiments with laboratory mice that adult neurogenesis is necessary for spatial memory formation (Snyder et al., 2005; Dupret et al., 2008; Clelland et al., 2009). At the same time, adult neurogenesis is absent in many bats (Chiroptera) (Amrein et al., 2007). Bats are foraging animals that fly long distances in search for food or to known sources of food. If adult neurogenesis is indeed required for the formation of spatial memory, one would expect robust adult neurogenesis in bats and its absence very surprising (Amrein et al., 2007). On the other hand, neurogenesis might be required only for the acquisition of cognitive skills needed for navigation/spatial separation. These skills could be acquired by bats at a young age and after that neurogenesis becomes irrelevant to their navigational abilities. Thus, the use of mice in experiments does not allow for us to distinguish the role of neurogenesis in the acquisition of cognitive skills required to perform tasks and the role of neurogenesis required to perform these tasks.

Cognitive neurogenesis is a development process and therefore, its most immediate and plausible therapeutic use can be expected in the field of intellectual developmental disorders. The use for the treatment of cognitive disorders and cognitive aspects of mental disorders could also be perspective. Cognitive enhancement and treatment of cognitive decline in the elderly might also be viewed as a perspective direction. At the same time, expectations need to be lowered that cognitive neurogenesis could serve as the source of new neurons and glial cells for brain repair and regeneration after traumatic brain injury, stroke, neurodegenerative diseases and other adverse events (Peng and Bonaguidi, 2018). Cognitive neurogenesis is supported by NTCs that are capable only of a limited production of neural precursors and therefore, it lacks intrinsic capacity for the neuron production sufficient for the extensive brain repair/restoration.

The continuation of cognitive neurogenesis in the adult mammalian brain shows that brain cognitive development relies not only on the modulation of synaptic connections in existing neuronal circuits but also requires changes of these circuits by incorporation of new neurons. Neurogenesis in the adult brain could be observed not only in mammals but in other vertebrate (Chapouton et al., 2007; Kempermann, 2015; Alunni and Bally-Cuif, 2016), showing that the requirement of neurogenesis for cognitive development might be a common trend in all vertebrate thus allowing us to conclude that only a continuously developing brain may properly adopt to a continuously changing world.

## Data Availability

All data generated or analyzed during this study are included in this published article.

## Author Contributions

MS contributed to the conceptualization of the study, the sources curation and analysis, the writing and preparation of the original draft, and the review, and editing of the manuscript.

### Conflict of Interest Statement

The author declares that the research was conducted in the absence of any commercial or financial relationships that could be construed as a potential conflict of interest.
